# Filtration-Histogram Based Magnetic Resonance Texture Analysis (MRTA) for the Distinction of Primary Central Nervous System Lymphoma and Glioblastoma

**DOI:** 10.3390/jpm11090876

**Published:** 2021-08-31

**Authors:** Claire L. MacIver, Ayisha Al Busaidi, Balaji Ganeshan, John A. Maynard, Stephen Wastling, Harpreet Hyare, Sebastian Brandner, Julia E. Markus, Martin A. Lewis, Ashley M. Groves, Kate Cwynarski, Stefanie C. Thust

**Affiliations:** 1 Neuroradiological Academic Unit, Department of Brain Repair and Rehabilitation, UCL Institute of Neurology, London WC1N 3BG, UK; john.alexander.maynard@gmail.com (J.A.M.); stephen.wastling@hns.net (S.W.); martin.lewis@alumni.ucl.ac.uk (M.A.L.); s.thust@ucl.ac.uk (S.C.T.); 2 Lysholm Department of Neuroradiology, National Hospital for Neurology and Neurosurgery, London WC1N 3BG, UK; ayisha.albusaidi@nhs.net; 3 Institute of Nuclear Medicine, University College London Hospitals NHS Foundation Trust, London NW1 2BU, UK; b.ganeshan@ucl.ac.uk (B.G.); ashley.groves@nhs.net (A.M.G.); 4 Imaging Department, University College London Hospitals NHS Foundation Trust, London NW1 3BG, UK; harpreet.hyare@nhs.net (H.H.); juliaemily.markus@nhs.net (J.E.M.); 5 Department of Neurodegenerative Disease, UCL Institute of Neurology and Division of Neuropathology, National Hospital for Neurology and Neurosurgery, London WC1N 3BG, UK; s.brandner@ucl.ac.uk; 6 Haematology and Oncology Department, University College London Hospitals NHS Foundation Trust, London NW1 3BG, UK; kate.cwynarski@nhs.net

**Keywords:** brain, lymphoma, glioblastoma, magnetic resonance imaging, computer-assisted

## Abstract

Primary central nervous system lymphoma (PCNSL) has variable imaging appearances, which overlap with those of glioblastoma (GBM), thereby necessitating invasive tissue diagnosis. We aimed to investigate whether a rapid filtration histogram analysis of clinical MRI data supports the distinction of PCNSL from GBM. Ninety tumours (PCNSL *n* = 48, GBM *n* = 42) were analysed using pre-treatment MRI sequences (T_1_-weighted contrast-enhanced (T_1_CE), T_2_-weighted (T_2_), and apparent diffusion coefficient maps (ADC)). The segmentations were completed with proprietary texture analysis software (TexRAD version 3.3). Filtered (five filter sizes SSF = 2–6 mm) and unfiltered (SSF = 0) histogram parameters were compared using Mann-Whitney U non-parametric testing, with receiver operating characteristic (ROC) derived area under the curve (AUC) analysis for significant results. Across all (*n* = 90) tumours, the optimal algorithm performance was achieved using an unfiltered ADC mean and the mean of positive pixels (MPP), with a sensitivity of 83.8%, specificity of 8.9%, and AUC of 0.88. For subgroup analysis with >1/3 necrosis masses, ADC permitted the identification of PCNSL with a sensitivity of 96.9% and specificity of 100%. For T_1_CE-derived regions, the distinction was less accurate, with a sensitivity of 71.4%, specificity of 77.1%, and AUC of 0.779. A role may exist for cross-sectional texture analysis without complex machine learning models to differentiate PCNSL from GBM. ADC appears the most suitable sequence, especially for necrotic lesion distinction.

## 1. Introduction

Primary central nervous system lymphoma (PCSNL) is a highly malignant brain tumour of lymphocyte origin, with the vast majority (>70%) histologically representing diffuse large B cell lymphoma [1]. Often, PCNSL occurs sporadically, but immunosuppression is a known risk factor and the incidence increases with age [2]. The reference standard treatment consists of an intense chemotherapy regime combining methotrexate, cytarabine, thiotepa, and rituximab (MATRix). MATRix substantially improves patient survival [3], but is associated with frequent and serious side effects, most notably bone marrow toxicity with neutropenia [4]. For this reason, securing a definitive diagnosis prior to commencing chemotherapy is vital.

Imaging has a central role in the investigation of PCNSL to identify, localise, and characterise brain lesions. Findings on anatomical MR imaging include single or multiple intracerebral masses involving the deep grey nuclei, corpus callosum, and/or periventricular region [5]. Typical PCNSL shows signals similar to grey matter on spin echo sequences and tend to exhibit avid, relatively homogenous gadolinium enhancement [6]. However, the lesion site, morphology, and contrast uptake patterns are all extremely variable [7]. The use of steroids for symptom control reduces lesion conspicuity on imaging and can render tissue results non-diagnostic. Thus, establishing the diagnosis of PCNSL can be a prolonged process, with a risk of jeopardising long-term outcomes [3].

A common imaging challenge is to differentiate PCNSL from glioblastoma (GBM), one of the most common malignant brain tumours in older populations [8]. While typical GBM features, such as necrosis, haemorrhages, and rim enhancement [9], differ from PCNSL morphology, each disease can mimic the other on conventional MRI sequences [10].

Diffusion-weighted MRI (DWI) is a physiological imaging technique, which functions on the presumption that low diffusivity reflects increased cellularity in neoplasia [11]. PCNSL commonly exhibits lower apparent diffusion coefficient (ADC) values than GBM [12,13,14], although ADC values are known to overlap [15,16,17] so that no clear threshold has been defined to distinguish the two entities. 

Machine learning methods are increasingly used as an adjunct for brain tumour characterisation in research. Numerous studies have proposed the use of feature extraction and radiomics models in PCNSL [18,19,20,21]. While their results are promising, such modelling techniques can be prone to “overfitting” and clinical translation remains challenging due to computational demand and the need for processing expertise. In contrast, filtration-histogram based MR imaging texture analysis (MRTA) is a workstation integrated software to examine tumour microstructure without the need for complex machine learning [22,23]. MRTA is based on the assumption that tissue properties are represented in the distribution of image pixel values, specifically heterogeneity [24], and it functions by drawing regions of interest onto an image with quantitative analysis initiated via mouse click. The application performs a filtration step, which serves to remove image noise, and extracts and enhances image features of different sizes before measuring signal intensity histogram parameters, which can be compared between regions or between groups of subjects [25]. The MRTA algorithm previously showed potential to identify glioma characteristics, including an ability for non-invasive genotyping [26,27]. The current study was performed to test whether the software would permit a distinction of untreated PCNSL and GBM using standard clinical MRI sequences.

## 2. Materials and Methods

### 2.1. Patient Cohort

**Figure 1:**
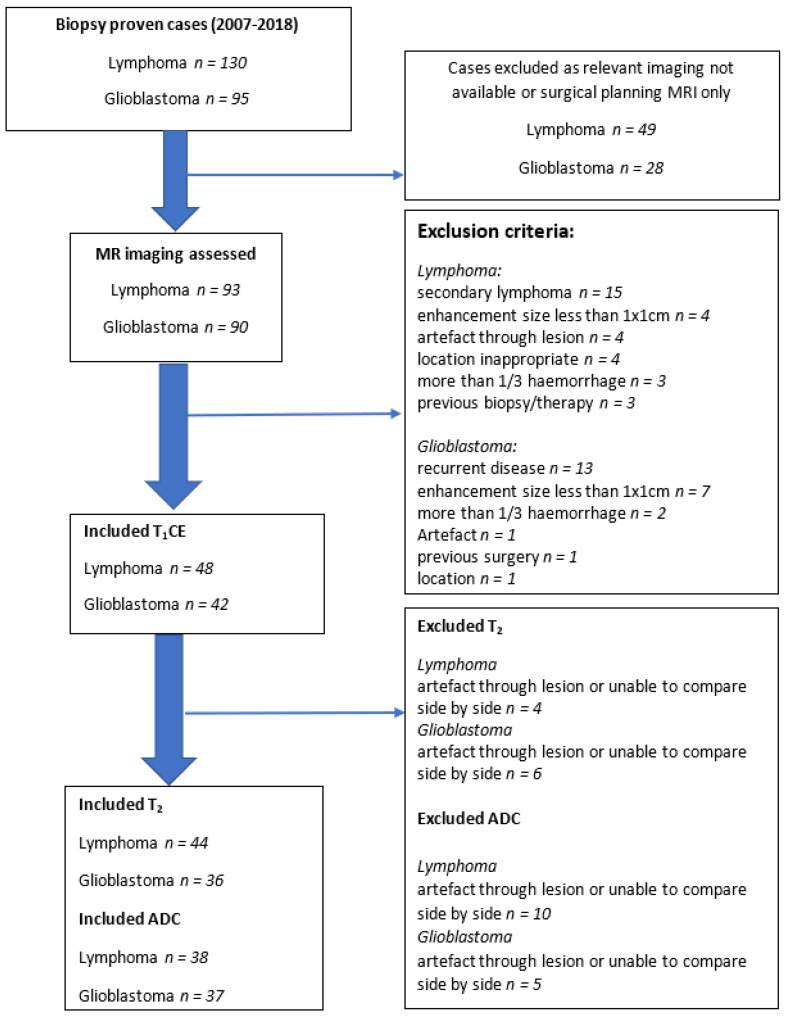
Flow diagram showing patient exclusion and inclusion criteria.

Institutional ethics review board approval was obtained, with informed consent waived for this retrospective imaging data study. Patients with a proven tissue diagnosis of PCNSL and glioblastoma between 2007 and 2018 were recruited from the neuropathology records. All 130 identified lymphoma cases and 95 randomly selected glioblastoma cases were assessed for the exclusion and inclusion criteria. Exclusion criteria were as follows: patients without pre-treatment gadolinium enhanced MRI scan available, surgical planning MRI sequences only, artefact obscuring the lesion or close vicinity, lesions obscured by large volume haemorrhage, secondary CNS (central nervous system) lymphoma, and previous treatment for CNS lymphoma. Inclusion criteria were: histological confirmation of PCNSL or glioblastoma and available treatment-naïve MRI imaging. T1-weighted post gadolinium (T_1_CE), T2-weighted (T_2_), and ADC maps were analysed, provided these were performed in the same setting to fulfil the requirement to be viewed side by side in the image software. Figure 1 shows a flow diagram of the patient selection for the study.

### 2.2. Tumour Segmentations

**Figure 2:**
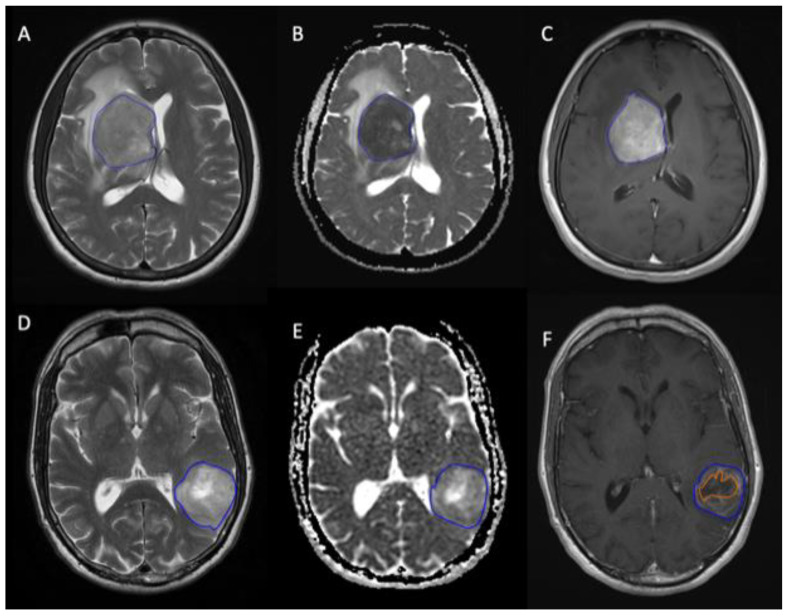
Example segmentations. T_2_, ADC maps, and T_1_CE images in two patients with PCNSL (**A**–**C**) and glioblastoma (**D**–**F**) demonstrating MRTA regions of interest (blue outlines). In the glioblastoma patient, the segmentation, excluding necrosis, is additionally shown (blue and orange outlines). ADC = apparent diffusion coefficient; PCNSL = primary central nervous system lymphoma; MRTA = magnetic resonance texture analysis.

For the patients fulfilling inclusion criteria, three pre-treatment MR imaging sequences (T_1_CE, T_2_, and ADC) were analysed. The imaging originated from 1.5T (*n* = 80) and 3T (*n* = 10) MRI scanners, and ADC maps were derived from b0 and b1000 imaging performed with three diffusion gradients. All image interactions were performed blinded to histological diagnosis. The segmentations were completed with proprietary texture analysis software (TexRAD version 3.3, TexRAD Ltd., www.texrad.com, part of Feedback Plc, Cambridge, UK; first accessed on April 2018) using the freehand drawing tool. For each individual, the largest tumour cross-section was selected by review of the T_1_CE sequence. Two different T_1_CE regions of interest (ROIs) were manually drawn: the first ROI incorporated the entire enhancing lesion inclusive of central necrotic components (*ROI_T1_a_*), and the alternative ROI non-enhancing components were excluded (*ROI_T1_b_*). Non-contrast imaging was inspected to verify the extent of tumour enhancement and to avoid ROI placement in haemorrhagic tumour components. For patients with available T_2_ and ADC maps, the *ROI_T1_a_* segmentation was directly copied onto the remaining sequences to derive *ROI_T2* and *ROI_ADC*, respectively. In cases where this was not possible due to significant differences in alignment, ROIs were manually redrawn on the T_2_ and ADC map with the T_1_CE sequence viewed side by side. To avoid partial volume effects during MRTA, only measurable lesions, defined as 10 × 10 mm^2^ or larger, were subjected to segmentation. Figure 2 shows an example of the segmentation technique. TexRad currently employs a multiple slice based (2D) segmentation method. From this, the software generates cumulative histograms from multiple slices prior to statistical analysis; therefore, the results are expected to be representative of a volumetric (3D) segmentation. Furthermore, it is likely that a fully implemented 3D segmentation (as compared to a multiple slice based 2D segmentation as implemented in this study) would not accurately delineate the lesion in a number of cases. This may require the operator to further manually edit and refine the segmentation on multiple slices. This would not only further increase intra-reader variability, but also be quite cumbersome and increase the processing time, which would be a further barrier to its adoption in routine clinical practice.

### 2.3. Texture Analysis (MRTA)

**Figure 3:**
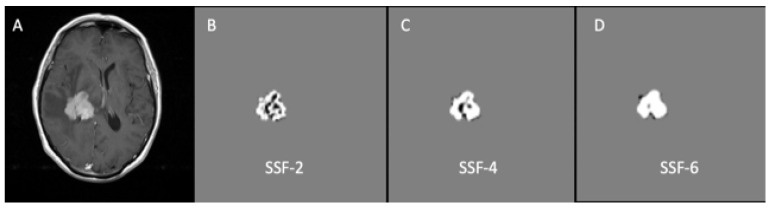
Example of the MRTA filtration process applied to a T_1_CE image (**A**) in a patient with PCNSL using fine (**B**), medium (**C**), and coarse filtration (**D**).

The technique employed in this study follows a previously published method [25,26]. The algorithm commences with a filtration step, which serves to remove image noise. This is performed for six spatial scale filter (SSF) values, whereby SSF = 0 corresponds to no filtration, SSF = 2 mm represents a fine texture scale, SSF = 3–5 mm signifies a medium texture scale, and SSF = 6 mm translates to a coarse texture scale (Figure 3). Following filtration, histogram and statistical parameters (mean, standard deviation, entropy, mean of positive pixels, skewness, and kurtosis) are automatically calculated for texture quantification. In cases where multiple contrast enhancing lesions were sufficiently large (10 × 10 mm^2^) to meet inclusion criteria, the ROI was drawn in the largest cross-section of each and the data was analysed as one combined lesion.

### 2.4. Visual Inspection

In addition to the computational analysis, two board certified neuroradiologists (ST, AAB) reviewed all available T_1_CE, T_2_ sequences, and ADC maps, blinded to diagnosis. For each case, the observers chose between a diagnosis of PCNSL, GBM, or assigned it to the category “uncertain”. A visual estimate was performed to quantify necrosis as detailed in https://wiki.cancerimagingarchive.net/display/Public/VASARI+Research+Project (Vasari MR Feature Guide v1.1, first accessed on 15 May 2018).

### 2.5. Statistical Analysis

All statistical testing was undertaken using SPSS version 25 (IBM, Armonk, NY, USA). Mann-Whitney U non-parametric testing was performed to compare T_1_CE, T_2_, and ADC map texture features of PCNSL and glioblastoma, with *p =* <0.05 considered significant. For each ROI type, a Mann-Whitney U test was used for five different filter sizes (SSF = 2–6) and for unfiltered images (SSF = 0) to assess the six MRTA parameters. In addition, subgroup analyses were performed to compare PCNSL and GBM histogram metrics for lesions with <1/3 necrosis and those with ≥1/3 necrosis. A Holmes correction for multiple statistical comparisons was applied for each set of analyses. For parameters that demonstrated a significant difference between the two tumour types, an assessment of the feature’s ability to discriminate the two lesion types was undertaken using a receiver operating characteristic (ROC) derived area under the curve (AUC) analysis.

## 3. Results

### 3.1. Patient Cohort

A total of 90 patients (*n* = 48 PCNSL and *n* = 42 GBM) fulfilled the inclusion criteria. The demographic results and lesion characteristics for the study population are presented in Table 1.

### 3.2. Analysis of All Tumours

The results for the T_1_CE, T_2_, and ADC segmentations of all tumours (*n* = 90) are provided in Table 2.

#### 3.2.1. All Tumours, ROI_T1_a_ and ROI_T1_b_

For *ROI_T1a* (PCNSL *n* = 48 and GBM *n* = 42), the histogram mean values differed significantly between PCNSL and GBM for all filter sizes (for SSF 2: U = 590, *p* = 0.001; SSF 3: U = 518, *p* < 0.0001; SSF 4: U = 475, *p* < 0.0001; SSF 5: U = 445, *p* < 0.0001; and SSF 6: U = 415, *p* < 0.0001). Specifically, *ROI_T1_a_* entropy values differed when using the smaller filter sizes (SSF 2: U = 573, *p* = 0.0004; and SSF 3: U = 603.5, *p* = 0.001), and skewness differed when unfiltered (U = 436, *p* < 0.0001). However, the maximal sensitivity (64.3%) and specificity (66.6%) for entropy were limited. For *ROI_T1_b_* (PCNSL N = 48 and GBM N = 42), skewness showed a significant difference between PCNSL and GBM unfiltered (U = 412, *p* < 0.0001), and kurtosis reached a significant difference for medium to coarse filter sizes (SSF 4: U = 516.5, *p* < 0.0001; SSF 5: U = 445.5, *p* < 0.0001; and SSF 6: U = 445, *p* < 0.0001). The largest *ROI_T1_b_* AUC was generated for coarse filtered kurtosis, translating to a sensitivity of 71.4% and specificity of 77.1% (AUC 0.779).

#### 3.2.2. All Tumours, T_2_ (ROI_T2)

*ROI_T2* (PCNSL *n* = 44 and GBM *n* = 36) demonstrated significant texture differences between PCNSL and GBM for mean histogram values (SSF 2: U = 346, *p* < 0.0001; SSF 3: U = 314, *p* < 0.0001; SSF 4: U = 298, *p* < 0.0001; SSF 5: U = 280, *p* < 0.0001; and SSF 6: U = 286, *p* < 0.0001) and entropy (SSF 2: U = 475, *p* = 0.002; SSF 3: U = 466.5, *p* = 0.002; SSF 4: U = 438.5, *p* = 0.001; SSF 5: U = 440.5, *p* < 0.001; and SSF 6: U = 456.5, *p* = 0.001) in all filter sizes, and for MPP (mean of positive pixels) using medium and coarse filter sizes (SSF 3: U = 463.5, *p* = 0.001; SSF 4: U = 444, *p* = 0.001; SSF 5: U = 413, *p* = 0.0002; and SSF 6: U = 399; *p* = 0.0001). The standard deviation and entropy differed for unfiltered images (SD: U = 414, *p* = 0.0002; and entropy: U = 372.65, *p* < 0.0001). Using a coarse filter size and mean T_2_ values, a sensitivity of 66.7% and specificity of 81.8% for identifying PCNSL were observed.

#### 3.2.3. All Tumours, ADC (ROI_ADC)

A significant difference was identified for the mean ADC measurement (PCNSL *n* = 38 and GBM *n* = 37. Unfiltered: U = 168, *p* < 0.0001; SSF 2: U = 329, *p* < 0.0001; SSF 3: U = 299, *p* < 0.0001; SSF 4: U = 276, *p* < 0.0001; SSF 5: U = 252, *p* < 0.0001; and SSF 6: U = 230, *p* < 0.0001), with PCNSL exhibiting generally lower ADC values than GBM. Additionally, the standard deviation (unfiltered: U = 263, *p* < 0.0001; SSF 2: U = 358, *p* = 0.0003; SSF 3: U = 378, *p* = 0.001; SSF 4: U = 371, *p* = 0.0004; SSF 5: U = 395, *p* = 0.001; and SSF 6: U = 405, *p* = 0.002), entropy (unfiltered: U = 326, *p* < 0.0001; SSF 2: U = 370.5, *p* = 0.0004; SSF 3: U = 385, *p* = 0.001; SSF 4: U = 396.5, *p* = 0.001; SSF 5: U = 398, *p* = 0.001; and SSF 6: U = 401, *p* = 0.001), and MPP (unfiltered: U = 168, *p* < 0.0001; SSF 2: U = 254, *p* < 0.0001; SSF 3: U = 298, *p* < 0.0001; SSF 4: U = 284, *p* < 0.0001; SSF 5: U = 322, *p* < 0.0001; and SSF 6: U = 314, *p* < 0.0001) differed between the two tumour types throughout all filter sizes. In addition, for skewness, a significant difference was evident when using a fine filter size (SSF 2: U = 369, *p* = 0.0004). The most distinctive result was shown for unfiltered ADC mean and MPP with a sensitivity of 83.8% and specificity of 78.9% (AUC 0.88) for identifying PCNSL.

### 3.3. Subgroup Analysis of Predominantly Solid (<1/3 Necrosis) Tumours

No significant (T_1_CE, T_2_, or ADC) differences were identified in the filtered histogram parameters for predominantly solid tumours (*n* = 46).

### 3.4. Subgroup Analysis of Partially Necrotic (≥1/3 Necrosis) Tumours

For the comparison of ≥1/3 necrotic contrast enhancing masses (*n* = 44), no significant differences in the T_1_CE (*ROI_T1_a_* and *ROI_T1_b_)* texture parameters were identified (PCNSL *n* = 8 and GBM *n* = 36). Table 3 shows the subgroup analysis results for the *ROI_T_2_* (PCNSL *n* = 8 and GBM *n* = 32) and *ROI_ADC* (PCNSL *n* = 5 and GBM *n* = 32) segmentations. A significant difference was demonstrated for the *ROI_T2* histogram mean values in medium to coarse filter sizes (SSF 4: U = 30, *p* = 0.001; SSF 5: U = 27, *p* = 0.001; and SSF 6: U = 26, *p* = 0.001), which reached a sensitivity of 71.9% and specificity of 87.5%. Using the ROI_ADC segmentations of partially necrotic tumours, PCNSL exhibited markedly lower MPP values than GBM, particularly when applying medium to coarse filters (SSF 4: U = 1, *p* = 0.0004, SSF 5: U = 7, *p* = 0.001, and SSF 6: U = 7, *p* = 0.001). With this, an AUC of 0.994 was observed, corresponding to a sensitivity of 96.9% and specificity of 100%.

### 3.5. Visual Rating

Table 4 presents the results of the consensus visual rating, which was generated through whole brain review using the combination of T_1_CE, T_2_, and ADC sequences available. Three tumours remained unrated due to observer uncertainty. For the remaining 87 tumours, the visual assessment produced a sensitivity of 97.82% and specificity of 92.68% for identifying PCNSL, with a positive predictive value (PPV) of 93.75% and negative predictive value (NPV) of 97.44%. If the unrated tumours were classed as incorrect diagnoses, the visual ratings translated to a sensitivity of 93.75% and a specificity of 90.48%, with a PPV of 91.84% and an NPV of 92.68% for recognising PCNSL.

## 4. Discussion

This study investigated the performance of a workstation-based tool for the rapid distinction of untreated PCNSL and GBM by free-hand segmentation of the largest tumour cross-section. Image texture differences between tumour types were apparent for T_1_CE and T_2_ imaging, but particularly for diffusion-weighted sequences (ADC maps).

If using the T_1_CE sequence alone, a moderately accurate (AUC 0.78) identification of PCNSL was achieved by confining regions of interest to solid enhancing tumour components. For the *ROI_T1_b_* method, kurtosis represented the most distinctive T_1_CE texture parameter, which can be explained through microscopic differences in tissue heterogeneity, as was previously observed for glioma genotyping using TexRad [27]. Kunimatsu et al. [28] recently demonstrated differences between PCNSL and GBM through the testing of 67 T_1_CE texture features in first and second statistical order modelling. Similar to our findings, heterogeneity parameters, such as entropy, permitted the correct diagnosis. Xiao et al. [29] recently applied MRTA to T_1_CE scans in PCNSL (*n* = 22) and GBM (*n* = 60), using a manually drawn whole lesion segmentation of lesions, unselected for the presence or absence of necrosis. Their group identified first order skewness as the best predictor of tumour type (AUC = 0.86), which is further indicative of differences in tumour heterogeneity.

In addition, our study shows an influence of the segmentation technique and lesion necrosis patterns on the discrimination of both tumour types. When analysing predominantly solid (<1/3 necrosis) masses alone, a T_1_CE based distinction was not achieved in our study, which could relate to the small subgroup numbers (*n* = 5 solid GBM) or the fact that we tested the performance of each image sequence as a standalone. Suh et al. [19] recently compared radiomic metrics combining T_1_CE, T_2_, and FLAIR images, and reported an accurate distinction (AUC 0.921) of solid PCNSL from GBM using a machine learning approach with a computational input of >6000 features. While this result is promising, a recent meta-analysis once more highlighted that machine learning algorithms for PCNSL versus GBM distinction tend to experience model overfitting, with reduced performance in subsequent testing [30].

It should be noted that published results for a radiomics-based distinction of PCNSL and GBM showed considerable variation, likely influenced by sequence choice and analysis methods. In their recent comparative study, Bathla et al. reported the highest accuracy for a combined use (AUC > 0.9) of ADC and T_1_CE, with or without FLAIR [31]. We note that this improved result was based on more complex machine learning models, which could impede rapid clinical translation when compared to the more intuitive, filtration-histogram-based texture analysis (via a workstation software such as TexRAD) employed in our study.

Early glioblastoma often shows solid or even minimal enhancement [32], which may correspond to greater tissue homogeneity at that time point. As such, the T_1_CE distinction between the two tumour entities could potentially be more challenging for early glioblastoma stages. Moreover, no significant T_1_CE texture differences were demonstrated between partially necrotic PCNSL and glioblastoma in our study, a question that, to the best of our knowledge, has not previously been investigated.

Across all (*n* = 90) tumours included in our research, ADC predicted the presence of PCNSL with the highest accuracy (AUC 0.88). This result is consistent with previous literature, with several studies reporting significantly lower ADC mean [17,33] and ADC minimum [15] values in PCNSL than glioblastoma. Using an ROI technique confined to solid tumour components, Wen et al. [15] previously observed differences between PCNSL and GBM in minimum ADC values (*n* = 39 PSCNL and *n* = 35 GBM), reporting a sensitivity of 74.5% and specificity of 74.1%. In a recent study by Bao et al., ADC histogram parameters showed no statistically significant difference, possibly due to patient numbers (*n* = 9 PCNSL versus 11 GBM) [34]. This difference in results could be due to patient numbers and/or method differences such as, for example, segmentation technique.

Our results furthermore suggest that ADC texture may facilitate the identification of necrotic PCNSL (AUC 0.994). The latter result could be explained by greater necrosis percentage or by differences in tissue architecture (e.g., cellularity), which may give rise to comparatively higher ADC values in GBM. To the best of our knowledge, we present the first study to specifically evaluate MRTA features in partially necrotic PCNSL; however, we did not further quantify necrosis (e.g., as a percentage) due to the small subgroup numbers. Similarly, the lack of a difference in the ADC texture between predominantly solid PCNSL and GBM may reflect the limited number of non-necrotic GBM recruited randomly (to avoid selection bias) in our study. The quantitative nature of an ADC map is advantageous for reproducibility, but further research will be required regarding the interpretation of this parameter with respect to tumour necrosis. In our research, the precision of a neuroradiologist visual inspection outperformed the MRTA software, provided that the experienced raters were permitted a review of the whole brain on multiple sequences.

This study has a number of limitations. The binary distinction of PCNSL from GBM served the purpose of trialing the accuracy of MRTA, but, in reality, more diverse differentials may apply in certain clinical situations or with atypical lesion morphology. We have not tested the reproducibility of segmentations for individual observers or time points, and further study will be necessary to reproduce the findings for a test population, ideally with a view to external validation. TexRAD software does not currently offer a dedicated (semi)automated segmentation function suitable for contouring brain lesions on MRI. A recognised limitation of manual tumour segmentation is observer dependence and time consumption, although good inter-observer concordance was demonstrated in recent glioma research [27,35]. Future studies should look at implementing better semi-automated segmentation and registration to minimise intra-reader variability. However, it is likely that the semi-automated, automated, or 3D segmentation would not accurately delineate the lesion in a number of cases. This may require the operator to further manually edit and refine the segmentation on multiple slices. This will not only further increase intra-reader variability, but also be quite cumbersome and increase the processing time, which will be a further barrier to its adoption in routine clinical practice.

## 5. Conclusions

This study identified several filtration-histogram-based texture features, most notably ADC parameters, which may support the distinction of PCNSL and glioblastoma on standard MRI sequences. The TexRad method could potentially complement an expert visual rating and appears clinically feasible as an easily operable, rapid workstation tool.

## Figures and Tables

**Table 1:** Study population demographics and tumour morphology.

Tumour Type		PCNSL	GBM	Total
Median (range) age in years		65 (20–84)	65 (34–81)	65.5 (20–84)
Gender (male/female)		22/26	24/18	46/44
<1/3 necrosis	T_1_CE	40 (83%)	6 (14%)	46
	T_2_	36 (90%)	4 (10%)	40
	ADC	33 (87%)	5 (13%)	38
≥1/3 necrosis	T_1_CE	8 (17%)	36 (86%)	44
	T_2_	8 (20%)	32 (80%)	40
	ADC	5 (14%)	32 (86%)	37

**Table 2:** MRTA analysis of all tumours (PCNSL *n* = 48 and GBM = 42).

**ROI_T1_a_**	**SSF**	**Mean**	**SD**	**Entropy**	**MPP**	**Skewness**	**Kurtosis**	**Sens/Spec (%)**	**ROI_T1_b_**	**SSF**	**Mean**	**SD**	**Entropy**	**MPP**	**Skewness**	**Kurtosis**	**Sens/Spec (%)**
	0	NS	NS	NS	NS	**0.784**	NS	28.6/39.6		0	NS	NS	NS	NS	**0.796**	NS	64.3/81.2
	2	0.293	NS	**0.716**	NS	NS	NS	64.3/66.6		2	NS	NS	NS	NS	NS	NS	NS
	3	0.257	NS	**0.701**	NS	NS	NS	**61.9/64.6**		3	NS	NS	NS	NS	NS	NS	NS
	4	**0.236**	NS	NS	NS	NS	NS	26.2/31.2		4	NS	NS	NS	NS	NS	**0.744**	69/68.7
	5	**0.221**	NS	NS	NS	NS	NS	33.3/27.1		5	NS	NS	NS	NS	NS	**0.779**	**71.4/77.1**
	6	**0.206**	NS	NS	NS	NS	NS	23.8/29.2		6	NS	NS	NS	NS	NS	**0.779**	64.3/77.1
**ROI_T_2_**	**SSF**	**Mean**	**SD**	**Entropy**	**MPP**	**Skewness**	**Kurtosis**	**Sens/Spec (%)**	**ROI_ADC**	**SSF**	**Mean**	**SD**	**Entropy**	**MPP**	**Skewness**	**Kurtosis**	**Sens/Spec (%)**
	0	NS	0.739	**0.765**	NS	NS	NS	63.9/68.2		0	**0.881**	0.813	0.768	**0.881**	NS	NS	**83.8/78.9**
	2	**0.782**	NS	0.7	NS	NS	NS	63.9/79.5		2	0.766	0.745	0.736	**0.819**	0.738	NS	70.3/71.1
	3	**0.802**	NS	0.705	0.71	NS	NS	66.7/79.5		3	0.787	0.731	0.726	**0.788**	NS	NS	64.9/71.1
	4	**0.812**	NS	0.723	0.72	NS	NS	69.4/79.5		4	**0.804**	0.736	0.718	0.798	NS	NS	73/76.3
	5	**0.823**	NS	0.722	0.74	NS	NS	**66.7/81.8**		5	**0.821**	0.719	0.717	0.771	NS	NS	70.3/73.7
	6	**0.819**	NS	0.712	0.75	NS	NS	**66.7/81.8**		6	**0.836**	0.712	0.714	0.777	NS	NS	70.3/73.7

Bold values represent the histogram feature with the highest area under the curve (AUC) for each filter value, with sensitivity and specificity at the optimal cut-off relating to this feature included. MRTA = magnetic resonance texture analysis; PCNSL= primary central nervous system lymphoma; GBM = glioblastoma; SSF = spatial scale filter; SD = standard deviation; NS = non-significant; MMP = mean of positive pixels; ROI_T1_a_ = region of interest for contrast enhanced T1 weighted image including necrotic regions; ROI_T1_b_ = region of interest for contrast enhanced T1 weighed image with necrotic regions excluded; ROI_T_2_ = region of interest for T2 weighted image; ROI_ADC = region of interest for apparent diffusion coefficient map.

**Table 3:** MRTA analysis of partially necrotic (≥1/3 necrosis) tumours (PCNSL *n* = 8, GBM = 36).

ROI_T2	SSF	Mean	SD	Entropy	MPP	Skewness	Kurtosis	Sens/Spec (%)	ROI_ADC	SSF	Mean	SD	Entropy	MPP	Skewness	Kurtosis	Sens/Spec (%)
	0	NA	NA	NA	NA	NA	NA	NA		0	NA	NA	NA	NA	NA	NA	NA
	2	NA	NA	NA	NA	NA	NA	NA		2	NA	NA	NA	NA	NA	NA	NA
	3	NA	NA	NA	NA	NA	NA	NA		3	NA	NA	NA	NA	NA	NA	NA
	4	**0.883**	NA	NA	NA	NA	NA	62.5/87.5		4	NA	0.956	NA	**0.99**	NA	NA	**96.9/100**
	5	**0.895**	NA	NA	NA	NA	NA	**71.9/87.5**		5	NA	NA	NA	**0.96**	NA	NA	78.1/100
	6	**0.898**	NA	NA	NA	NA	NA	**71.9/87.5**		6	NA	NA	NA	**0.96**	NA	NA	78.1/100

Bold values represent the histogram feature with the highest area under the curve (AUC) for each filter value, with sensitivity and specificity at the optimal cut-off relating to this feature included. MRTA = Magnetic resonance texture analysis; PCNSL = primary central nervous system lymphoma; GBM = glioblastoma; SSF = spatial scale filter; SD = standard deviation; NS = non-significant; MMP = mean of positive pixels. ROI_T_2_ = region of interest for T2 weighted image; ROI_ADC = region of interest for apparent diffusion coefficient map.

**Table 4:** Neuroradiologist whole brain visual assessment using T_1_CE images, T_2_ images, and ADC maps.

		Tissue Diagnosis	
		PCNSL	Glioblastoma
Rater diagnosis	PCNSL	45	3
	Glioblastoma	1	38
	Unrated	2	1

T_1_CE = contrast enhanced T1 weighted image; ADC = Apparent diffusion coefficient; PCNSL = primary central nervous system lymphoma.

## Data Availability

Data supporting results are not publicly available for this study, but may be obtained from the authors upon request.
